# Alveolar Adenoma: A Rare Benign Tumour of the Lung with A Challenging Diagnosis

**DOI:** 10.5146/tjpath.2021.01547

**Published:** 2022-05-19

**Authors:** Soumaya Graja, Saadia Makni, Abdessalem Hentati, Chiraz Chaari, Tahya Sellami-Boudawara, Rim Kallel

**Affiliations:** Department of Pathology, Habib Bourguiba University Hospital, Sfax, Tunisia; Department of Cardiothoracic Surgery, Habib Bourguiba University Hospital, Sfax, Tunisia

**Keywords:** Lung tumour, Alveolar adenoma, Immunohistochemistry, Differential diagnosis

## Abstract

Alveolar adenoma is a rare lung benign tumour originating from type II pneumocytes. It presents as a well-defined nodule. In some cases, it is difficult to differentiate from lung cancer. Few cases of this tumour have been reported. We describe here a case of alveolar adenoma in a 63-year-old man discovered incidentally on chest X-ray. The lesion was reported as lepidic adenocarcinoma in bronchoscopic biopsy. The patient underwent a thoracoscopic left lower lobectomy. The histopathological and immunohistochemical examinations resulted in a diagnosis of alveolar adenoma. We report this case to describe its morphological and immunohistochemical characteristics and to emphasize its diagnostic difficulties.

## INTRODUCTION

Alveolar adenoma (AA) is a rare lung neoplasm, accounting for less than 1% of all lung tumours. It represents one of several types of pulmonary adenomas recognized by the revised World Health Organization classification of lung tumours (1). There are only forty cases reported in the English medical literature.

It has a particular histology and it consists of multi-cystic spaces lined by an alveolar epithelium overlying a spindle cell rich stroma (2). It is typically more common in middle-aged women and presents as an asymptomatic mass found in chest radiographs (3). The purpose of this report is to describe a rare example of AA, and to discuss the differential diagnosis with bibliographical considerations.

## CASE REPORT

**Figure 1:**
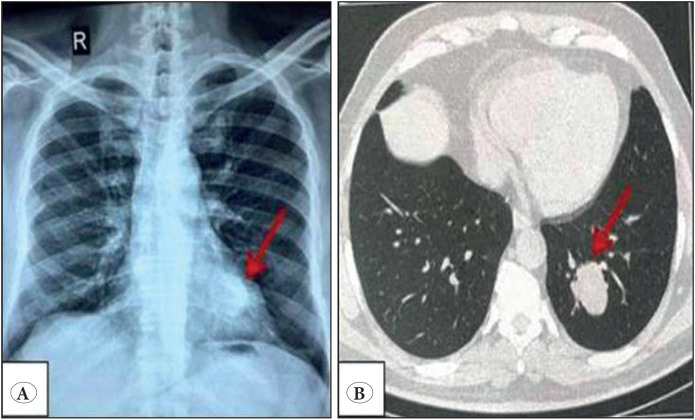
Chest X-ray **(A)** and CT scan **(B)** present a 33 mm, well-circumscribed, homogenous, non-calcified, solitary nodule in the left lower lobe.

**Figure 2:**
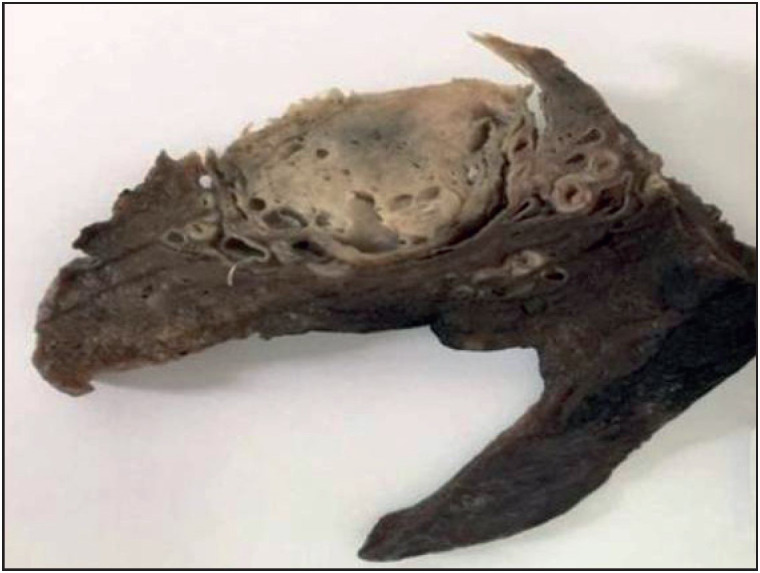
Macroscopic view of the mass: a well-circumscribed encapsulated tumor with spongy appearance and grey-white cut surface.

A 63-year-old man, without a significant past medical history, presented with a solitary pulmonary nodule located in the left lower lobe of the lung with a diameter of 33 mm that was incidentally discovered on chest X-ray imaging (Figure 1A). Bronchoscopic biopsy of the mass was performed and the microscopic examination suggested a lepidic adenocarcinoma. The patient underwent a thoracoscopic lobectomy. Macroscopic examination revealed a well-circumscribed encapsulated peripheral tumor measuring 4×3,3×2,8 cm. The cut surface was grey-white with a spongy appearance (Figure 2).

**Figure 3:**
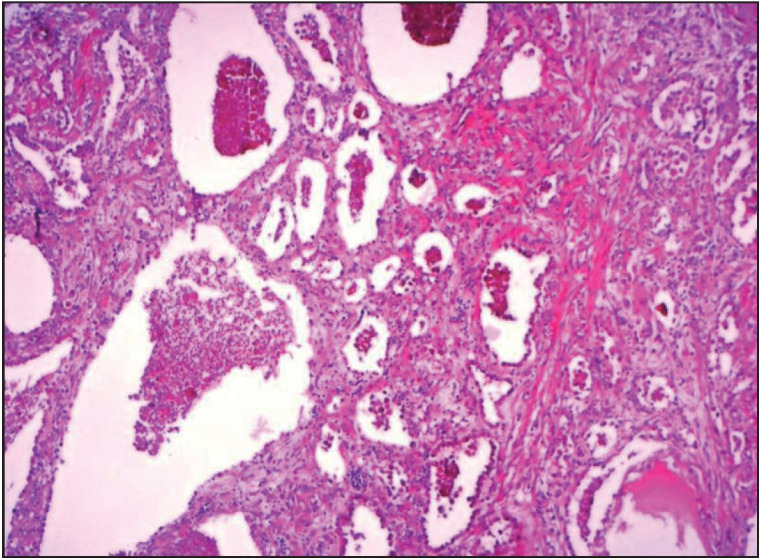
Multi-cystic lesion with ectatic spaces filled with eosinophilic granular material (H&E, 50x).

**Figure 4:**
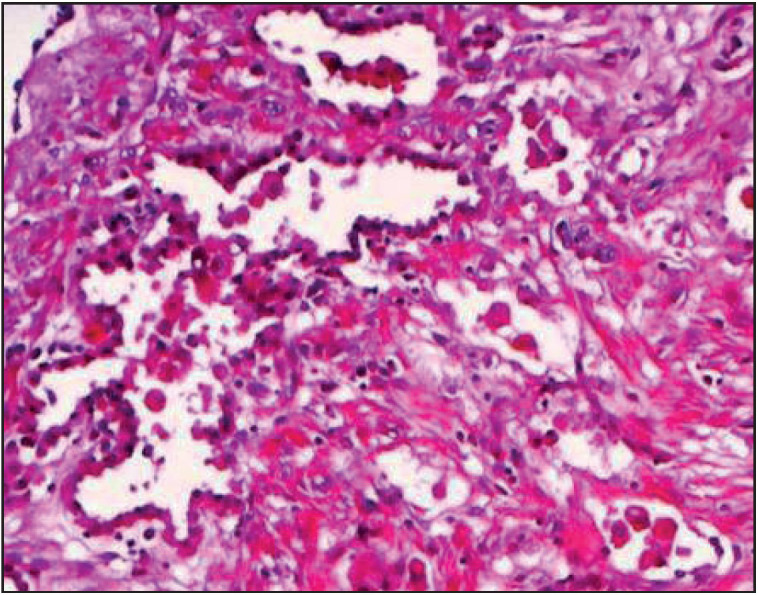
The cystic spaces are variable in size, and are lined with thin cuboidal alveolar epithelium (H&E, 100x).

Histologically, the tumor was a multicystic mass easily distinguished from the surrounding lung parenchyma (Figure 3). The cystic spaces were variable in size and were lined with thin cuboidal alveolar epithelium with bland cytology, and they were focally filled with granular eosinophilic material (Figure 4). The mid-alveolar septa were flattened with microvessels and few spindle-shaped cells, with local mild chronic inflammation. No cellular atypia, necrosis, invasion of adjacent parenchyma, or vascular invasion was seen. The adjacent lung tissue was normal.

**Figure 5:**
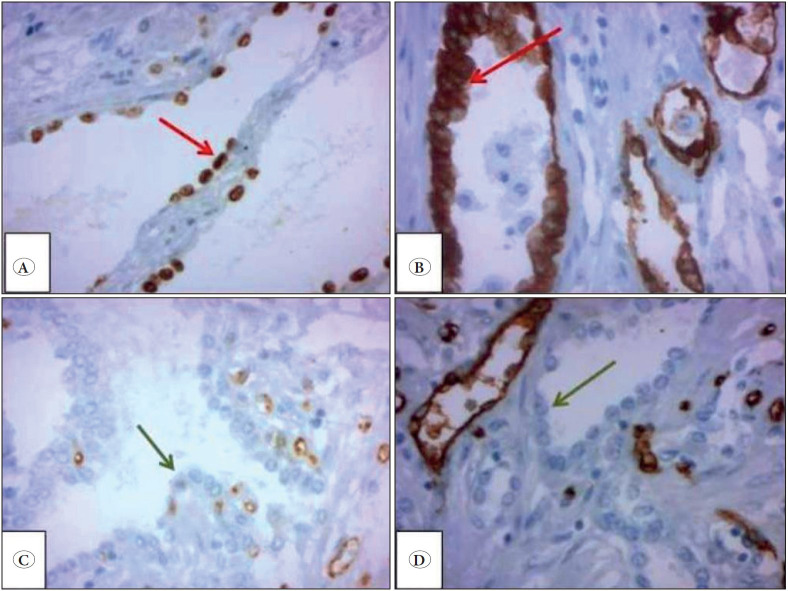
The epithelial lining cells are positive for TTF1 **(A)**, CK **(B)**, and are negative for CD31 **(C)** and CD34 **(D)** (H&E, 400x).

In the immunohistochemical analysis, the epithelial cells lining the spaces were diffusely positive for thyroid transcription factor-1 (TTF-1), and cytokeratin (CK); but were negative for clusters of differentiation 31 (CD31), and 34 (CD34) (Figure 5A-D). The Ki-67 proliferation index was low.

The patient was well with no recurrence or metastasis at the 1-year follow up evaluation.

## DISCUSSION

Alveolar adenoma (AA) of the lung is a very rare benign neoplasm. It was reported for the first time by Yousem and Hochholzer in 1986 (2). In most cases, it occurs in middle-aged and older women, with an age range from 39 to 74 years, and with a mean of 54 years (4).

The histogenesis of this tumour remains unknown. The majority of authors admit that type II pneumocytes are the origin of the epithelial component (4,5). Some authors, however, have proposed that the cell of origin in AA is probably a primitive mesenchymal cell differentiated toward a type II pneumocyte.

Typically, AA has no clinical manifestations (3,6). Rarely, the patient can present for chest pain, shortness of breath, and persistent cough (3). Commonly, AA is discovered by coincidence on chest X-ray imaging. It presents as a solitary well-demarcated homogenous mass. The left lower lobe is the preferential site; however, it can arise in any other lung lobe.

Macroscopic examination shows a subpleural or parenchymal spongy mass that is well demarcated from the underlying lung parenchyma (2). It can occur in any lobe, and is located underneath intact pleura.

Histologically, these tumours have a multicystic appearance. The cysts are lined with a single layer of flattened or cuboidal epithelial cells. The cystic lumina are usually filled with histiocytes, erythrocytes, and periodic acid-Schiff-positive granular material (2,6). The interstitial component usually comprises collagen fibrils, spindle cells, or modified smooth muscle cells. Lymphocytes, mast cells and eosinophils can be seen in the interstitial areas (2,6,7).

In all reported cases, epithelial lining cells were positive for TTF-1, CK, surfactant apoprotein A, and Napsin A which confirms that the lining epithelial cells are type II pneumocytes, and they are negative for protein S100, and Ki67 (4,8).

The diagnosis of AA is extremely difficult when only small tissue samples are available or on frozen sections. In these cases, AA could erroneously be diagnosed as malignant tumour or be considered as normal lung parenchyma (7).

Papillary adenoma, sclerosing hemangioma, atypical adenomatous hyperplasia, lepidic adenocarcinoma, lymphangioma, and hamartoma are in the differential diagnosis of AA (3,7).

Papillary adenoma is also asymptomatic and appears as an isolated peripheral nodule on chest-X ray. The difference with AA is that papillary adenoma shows an abundant papillary structure lined with epithelial cells resembling type II pneumocytes, Clara cells, or ciliated cells. Sclerosing hemangiomas have the same clinical presentation as AA, but are histologically distinctive by the association of four architectural components; papillary, solid, vascular and sclerotic. The TTF-1 expression observed in AA is very important to differentiate AA from sclerosing hemangiomas. Lymphangioma and AA have comparable histologic features. Only an immunohistochemical study can differentiate them. Cells lining the cystic spaces in lymphangioma are positive for factor VIII and CD31, and negative for CK (7).

The presence of a lepidic pattern, cellular atypia, mitosis, and a high Ki-67 proliferation index could aid to distinguish AA from lepidic adenocarcinoma (6). The indolent clinical progression, the absence of recurrence and metastasis after complete resection are important to distinguish AA from adenocarcinoma of the lung. Confirming the diagnosis on small biopsy sections can be difficult. This was the case for our patient. The final diagnosis of AA was made after complete resection. Immunohistochemical study is not specific. AA cells are positive for TTF-1, and almost all of them are CK positive; they show a low Ki-67 proliferation index. In accordance with these findings, our case demonstrated positivity for TTF-1, and CK and the Ki-67 proliferation index was low.

AA can be mistaken for hamartoma. However, this lesion is typically composed of a mixture of cartilage, fat, smooth muscle and bronchiolar tissue (7).

AA is a benign tumour, and complete excision is considered curative without the need for additional treatment. To the best of our knowledge, recurrence or metastases have not been reported (6).

In conclusion, AA presents as an incidental solitary pulmonary mass. It is entirely benign. AA is a difficult lesion to diagnose especially on small biopsy specimens. It is considered as a differential diagnosis of a solitary pulmonary nodule. We report this case because of its rarity and good prognosis, and to avoid a misdiagnosis, especially with adenocarcinoma.

## Conflict of Interest

The author(s) declared no potential conflicts of interest with respect to the research, authorship, and/or publication of this article.

## Funding

The author(s) received no financial support for the research, authorship, and/or publication of this article.
